# 

**Published:** 1920-01-31

**Authors:** 


The Right Hon. the Lord Mayor of London has consented to act as Chairman of the General Committee Promoting Health Week, to be held from May 2 to May 8, 1920.

### PASSING EVENTS AND TOPICS.

### Death of Mr. W. H. Pearce.

WE regret to record the death of Mr. William Henry Pearce, which occurred on the 12th inst. Mr. Pearce was connected with Paddington Green Children's Hospital throughout its history, having been collector and clerk when the institution was a dispensary in Bell Street, and secretary from the date of the move to Paddington Green, where the permanent building was opened in 1895. Mr. Pearce retired in 1917 after thirty-four years' service. He was very popular in the hospital world, and at his funeral on Saturday last, at Hammersmith Cemetery, such bodies as the Nosocomia Lodge of Freemasons, the Hospital Officers' Club, and the Incorporated Society of Hospital Officers were represented.

### The Story of "Bart's."

THE story of "Bart's" was told by Mr. A. H. Blake, M.A., F.R.Hist.S., to the London Rambling Society in the course of a delightful excursion to Smithfield the other day. Visiting the famous old church, the members were surprised to hear how recently the north transept was used as a blacksmith's forge, and were delighted with the thoroughness of the restoration of the beautiful Norman work. The tomb of Rahere, the founder of "Bart's," is finely preserved. Once thieves broke it open, but found nothing of value. During the air raids a window was discovered that had been blocked up for 350 years. The Rambling Society has visited several famous hospitals, and we are asked to say that any who would like to join in its weekly excursions can receive full syllabus on application to the Secretary, 36 Basinghall Street, E.C.

### Great Northern Central Hospital.

ONE HUNDRED AND FIFTY pupils of the Tollington Park Central and Montem Street Girls' Schools were entertained at the Great Northern Central Hospital, Holloway, on January 20 by the North Division of the League of the Roses, in recognition of the support received from them. Last year the League contributed £1,700 to the hospital.

### Hospital and Institutional Results.

**Wolverhampton and Staffs General Hospital.**—The hospital received £7,896 from the Hospital Saturday Fund and £1,526 from "Alexandra Day." The annual subscriptions for the year amounted to £3,118, the largest amount in any year since the foundation of the hospital.

**Cancer Hospital.**—The excess of ordinary expenditure over income in 1918 was £13,397, but legacies for general purposes amounting to £17,810 are not included in the income and expenditure account. The working expenses of the Cancer Hospital Research Institute, £2,537, are included in extraordinary expenditure.

**West Kent General Hospital**—The present building and equipment being inadequate, the committee are considering what should be done in order to put the hospital on a proper basis to meet the ever-growing demands. There was a surplus of £1,364 last year.

**Cumberland and Westmorland Mental Hospital.**—The severe epidemic of influenza sent nine cases to this mental hospital. Of seventeen mental service cases seven showed no cause beyond war experiences.

**Wilts County Asylum.**—The main incident of the year was the reception at very short notice of nearly 200 patients from the Portsmouth Borough Asylum in July, consequent on that asylum being taken over by the American military authorities. The difficulties and dangers attending such overcrowding are such that the committee would not have accepted them had not the matter been represented to them as of the most urgent national importance.

**James Murray's Royal Asylum, Perth.**—Once again it is interesting to note what has been the almost universal observation elsewhere, that the war-time conditions of the greater part of the year have had little influence on the cases admitted to the institution. It would, however, be a matter causing no great surprise if, in this succeeding period of relaxation and general unrest, there was found a decided increase in the incidence of mental disease.

**Royal Hospital and Home for Incurables, Putney.** During the year (which at Putney ends on September 30) the name of the institution has, with the approval of the King, been changed from "Royal Hospital" to "Royal Hospital and Home," and a Royal Charter of Incorporation has been granted.

**The Royal Surgical Aid Society.**—The income of the Society for the year ending September 30, 1919, was £33,625, and £27,175 of this amount was spent on surgical appliances and the necessary fees of surgeons and fitters. 18,979 cases were relieved during the financial year.

**Croydon General Hospital.**—The hospital year ends on June 30. The accounts are so unlike the uniform system that it is too difficult in a short review to refer to them, but it is stated that the total income was £18,748, and the total expenditure £10,271.

### THE COLLEGE OF NURSING (Limited by Guarantee).

### LONDON CENTRE.

Miss E. Collins, a member of the Stobart Unit, gave on Saturday, January 24, 1920, a vivid account of her experiences during the retreat in Serbia. She kept her audience deeply interested for two hours, for the hearing of such experiences from one who herself participated in almost incredible hardships brought to some a realisation of the colossal difficulties which had been met and overcome by many of their sister-nurses. So successful was the lecture that the London Centre hopes to arrange for Miss Collins to repeat it, illustrated by lantern slides. A larger room will then be engaged in order to enable all of the members to have the opportunity of attending.

After the lecture the meeting resolved itself into a social gathering, tea, provided by the Executive Committee of the London Centre, being served in the Club rooms.

The first (postponed) lecture on "Public Speaking" will be held on Thursday, February 12, at 8 p.m., at the Medical Society's Rooms, 11 Chandos Street, W. 1.

### LIVERPOOL CENTRE.

All phases of Japanese life, scenery, and flowers were shown on the screen at the interesting lecture on Japan given on the 21st inst. in the New Arts Theatre of the Liverpool University by Dr. Murray Cairns. The photographs, which had all been taken and exquisitely coloured by the Japanese, were highly appreciated by the audience. So keen was the enjoyment of those who were present that many have since written to the Hon. Secretary to express their pleasure.

The next lecture will be held on February 4 in the lecture theatre of the Royal Infirmary, when Miss Eleanor F. Rathbone will speak on "The Civic Privileges and Duties of the Woman Citizen."

### The Book Market.

Lea and Feliger, Philadelphia: "Modern Urology." By Hugh Cabot, M.D., F.A.C.S. $14 per set.

Oxford Medical Publications: "The Future of Medicine." By Sir James Mackenzie. 8s. 6d. net.

J. Wright and Sons: "National Health." By Ferdinand Rees, M.D. 1s. 6d. net.

P. S. King and Son, Ltd.: "The Education Act, 1918." By Arthur A. Thomas, M.B.E., B.A. 6s. net.

John Bale, Sons and Danielsson, Ltd.: "Facial Neuralgia and its Treatment." By J. Hutchinson, F.R.C.S. 15s. net.

Bhuvaneshvari Ashram Bahadurganj, Allahabad. The Panini Office, Allahabad, India: "Diabetes and its Dietetic Treatment." By B. D. Basu. Rs. 1.8.

Eveleigh Nash: "A Plea for the Treatment of Cancer Without Operation." By R. Bell, M.D. 2s. 6d. net.

H. K. Lewis: "Elementary Organic Chemistry." By F. Pilkington Sargeant, Ph.C., F.C.S. 4s. net.

Harrison and Sons: "The Industrial Council for the Building Industry." 1s. net.

Cassell and Co.: "The Welfare of the School Child." By Joseph Cates, M.D., D.P.H. 5s. net.

J. Wright and Sons: "Pye's Elementary Bandaging and Surgical Dressing." 14th edition. Revised. By V. Zachary Cope, B.A., M.D. 3s. 6d. net.

Chapman and Hall: "Food. Its Composition and Preparation." By Mary T. Dowd and Jean D. Jameson. 6s. net.

Scientific Press, Ltd.: "Change of Life: Its Difficulties and Dangers." By Dr. Mary Scharlieb. 1s. 3d. net.

C. and S. Livingstone: "Diseases of the Eye." By Wm. Geo. Sym, M.D. 1s. 6d. net.

"Physics." Part I. (Second edition.) 1s. 6d. net.

"Half a Century of Small-pox and Vaccination." By John C. McVail. 5s. 6d. net.

### MATRONS' AND SISTERS' APPOINTMENTS.

ROYAL INFIRMARY, BLACKBURN.—Miss Gladys Thwaite has been appointed sister. She was trained at Hull Royal Infirmary, where she was later ward sister and night superintendent. She has since been sister at the Victoria Nursing Home, Hull.

STAMFORD AND RUTLAND INFIRMARY.—Miss Ethel M. Wilson has been appointed sister. She was trained at Firvale Infirmary, Sheffield, and at the South-Eastern Fever Hospital, and has since been ward sister at Monsall Fever Hospital, at Norwich Fever Hospital, and at Ladywell Sanatorium, Salford.

BRENTWOOD, HIGH WOOD, ESSEX.—Miss Emma Field has been appointed sister. She was trained at Selly Oak Infirmary, Birmingham, and has since been staff nurse at Kingston-on-Thames Infirmary, ward sister at the Royal National Hospital for Consumption, Ventnor, I.O.W., and under the Territorial Force Nursing Service at the 4th Southern General Hospital, Plymouth, and at Malta.

MANCHESTER CITY MATERNITY AND CHILD WELFARE CENTRE.—Miss Clarke has been appointed assistant superintendent. She was trained at Bagthorpe Infirmary, Nottingham, and has since been surgical sister at Tranmere Infirmary, Birkenhead, staff midwife at Plaistow District Training School, head nurse at Hemsworth Infirmary, and matron of Urmston Cottage Hospital. She holds the C.M.B. certificate.

PATRICROFT INFIRMARY, GREEN LANE.—Miss Ann Dolan has been appointed superintendent-nurse. She was trained at West Derby Union Institution, where she was later staff nurse, sister, theatre sister, and night superintendent.

BOLTON INFIRMARY AND DISPENSARY.—Miss Winifred Thorn has been appointed sister. She was trained at Essex County Hospital, where she was later sister of the men's surgical ward and night sister. She has since been sister of the male ward and out-patients' sister at Bury St. Edmunds Hospital.

BOOTH HALL INFIRMARY FOR CHILDREN, BLACKLEY, NEAR MANCHESTER.—Miss Ethel Ashton has been appointed matron. She was trained at Crumpsall Infirmary (Manchester Union), where she was later first assistant superintendent of nurses.

BIRKENHEAD AND WIRRAL CHILDREN'S HOSPITAL.—Miss Alice Cowgill has been appointed sister. She was trained at the General Hospital, Barrow-in-Furness, was later staff nurse at the Keighley War Hospital, and theatre and ward sister at the Military Hospital, Wrexham, and at the B.R.C.S. Hospital, Ashton-under-Lyne.

